# Comparative Clinical and Functional Profiles of Monosymptomatic and Non-monosymptomatic Primary Enuresis in Children

**DOI:** 10.7759/cureus.111200

**Published:** 2026-06-20

**Authors:** Karima Ould Said, Mohammed Amine Merbouh, Khadidja Benallal, Abla Chalabi-Benabdallah

**Affiliations:** 1 Faculty of Medicine Taleb Mourad, Djillali Liabès University of Sidi Bel Abbès, Sidi Bel Abbès, DZA; 2 Department of Pediatrics, Hassani Abdelkader University Hospital, Sidi Bel Abbès, DZA; 3 Department of Epidemiology and Preventive Diseases, Hassani Abdelkader University Hospital, Sidi Bel Abbès, DZA; 4 Department of Pediatrics, University of Oran 1 - Ahmed Ben Bella, Oran, DZA

**Keywords:** bladder dysfunction, children, enuresis, monosymptomatic enuresis, nocturnal polyuria, non-monosymptomatic

## Abstract

Introduction

Primary nocturnal enuresis is a common pediatric condition, classified into monosymptomatic (MPE) and non-monosymptomatic (NMPE) based on the presence of daytime lower urinary tract symptoms (LUTS). Distinguishing between these two forms is essential, as it directly influences management strategies. This study aimed to compare the clinical, functional, and psychological characteristics of children with MPE and NMPE.

Materials and methods

A comparative observational study was conducted over two years, including children aged five to 15 years who presented with primary nocturnal enuresis. Clinical evaluation was performed using a structured questionnaire and a seven-day voiding diary. Functional bladder capacity (FBC) and nocturnal urine production were assessed. Patients were classified as MPE or NMPE according to the International Children's Continence Society criteria.

Results

A total of 171 children were included, of whom 86 (50.3%) were classified as MPE. The NMPE group was significantly younger than the MPE group (7.36 ± 2.02 vs. 9.12 ± 2.36 years, t = 5.19, p < 0.001). A significant male predominance was noted in the MPE group (67.4% vs. 44.4%, χ² = 8.97, p = 0.003; Cramér’s V = 0.23). Nocturnal enuresis severity was higher in the MPE group, with 56.9% of children having 3 or more wet nights per week, whereas 67.1% of children in the NMPE group had fewer than one wet night per week (χ² = 85.67, p < 0.001; Cramér’s V = 0.71). FBC was significantly reduced in the NMPE group (χ² = 120.15, p < 0.001; Cramér’s V = 0.84). In contrast, MPE showed a strong association with nocturnal polyuria (59.3% vs. 1.2%; χ² = 68.25, p < 0.001; Cramér’s V = 0.62). Both groups experienced a considerable psychological impact, with no significant differences noted between them.

Conclusions

NMPE represents a more complex bladder dysfunction phenotype, whereas MPE appears to be primarily associated with nocturnal urinary overproduction. Both phenotypes were associated with substantial psychosocial burden. Differentiating these subtypes is essential for individualized management strategies.

## Introduction

Primary nocturnal enuresis is defined as involuntary urination during sleep in children older than five years in the absence of identifiable organic, neurological, or urological abnormalities [1]. It represents one of the most common functional urinary disorders in the pediatric population and remains a frequent reason for consultation in both primary care and specialized pediatric urology settings [1,2].

The International Children’s Continence Society (ICCS) classifies enuresis into two major subtypes: monosymptomatic primary enuresis (MPE), characterized by the absence of daytime lower urinary tract symptoms (LUTS), and non-monosymptomatic primary enuresis (NMPE), in which nocturnal enuresis is associated with daytime urinary symptoms such as urgency, increased frequency, or daytime incontinence [1,2]. This distinction is not merely descriptive; it reflects distinct underlying pathophysiological mechanisms and has direct implications for therapeutic strategies.

The prevalence of enuresis varies with age, affecting approximately 5 to 10% of school-aged children, with a spontaneous annual resolution rate estimated at 15% [3]. Despite this tendency toward spontaneous improvement, a substantial proportion of children continue to experience persistent symptoms that may significantly affect psychological well-being and family dynamics. Enuresis is frequently associated with low self-esteem, social withdrawal, anxiety, and increased parental stress [4,5].

The pathophysiology of enuresis is multifactorial and involves three principal mechanisms: nocturnal polyuria, reduced functional bladder capacity (FBC), and impaired arousal response during sleep [6,7]. These mechanisms may coexist in varying proportions among patients. MPE is typically associated with nocturnal polyuria and altered circadian vasopressin secretion, leading to increased nighttime urine production. In contrast, NMPE is more frequently associated with bladder dysfunction, including detrusor overactivity and decreased bladder capacity [8,9].

Bladder-bowel dysfunction has also emerged as an important contributing factor, particularly in NMPE. Constipation may influence bladder function through mechanical and neurophysiological mechanisms, leading to increased bladder instability and urinary symptoms [10,11].

Despite advances in understanding enuresis, distinguishing between monosymptomatic and non-monosymptomatic enuresis remains challenging in routine clinical practice. In real-world settings, overlapping symptoms and variability in bladder function may complicate classification [12].

A better characterization of these subtypes is essential, as it directly influences therapeutic strategies and prognosis. Therefore, this study aimed to provide a comprehensive comparison of clinical, functional, and psychosocial profiles of children with MPE and NMPE in a real-life clinical setting.

## Materials and methods

Study design and setting

This was a comparative observational study conducted over a two-year period (2017-2018) at the Department of Pediatrics, Hassani Abdelkader University Hospital Center, Sidi Bel Abbès, Algeria.

Study population

All eligible children presenting for evaluation of primary nocturnal enuresis during the study period were consecutively enrolled (Table 1).

**Table 1:** Inclusion and exclusion criteria

Inclusion criteria	Exclusion criteria
Children aged 5 to 15 years	Secondary enuresis
Primary nocturnal enuresis	Neurological disorders
No prior treatment affecting bladder function	Congenital urological abnormalities
Available clinical and functional data	Urinary tract infection
Informed consent obtained	Diabetes mellitus or insipidus

Patients were classified into two groups according to ICCS criteria [1,2]: MPE was defined as the absence of daytime LUTS, whereas NMPE was defined by the presence of daytime LUTS.

Clinical and functional assessment

All patients underwent a standardized evaluation, including demographic data (age, sex), urinary symptoms (urgency, frequency, daytime incontinence), frequency of enuresis episodes, history of urinary tract infections, and assessment of constipation. A seven-day voiding diary was used to collect objective functional data, including daytime voiding frequency, maximum voided volume, and nocturnal urine production.

Definitions

Estimated nocturnal urine output was calculated as the sum of nocturnal urine losses, including the first morning void and the weight of wet diapers (1 g = 1 mL). FBC was defined as the maximum voided volume excluding the first morning void, recorded during the seven-day diary period.

Estimated bladder capacity (EBC) was calculated using Koff’s formula: (age in years + 1) × 30 mL [13]. Normal FBC was defined as 65-150% of the EBC. Reduced bladder capacity was defined as a maximum voided volume of <65% of the EBC. Nocturnal polyuria was defined as nocturnal urine output exceeding 130% of the EBC [2].

Constipation was defined according to Rome III criteria for functional constipation in children, requiring at least two of the following symptoms for at least one month: infrequent bowel movements, painful defecation, fecal incontinence, or stool-withholding behavior [14].

Psychosocial impact was evaluated through semi-structured clinical interviews conducted by trained clinicians. These interviews explored emotional and social dimensions, including embarrassment, social withdrawal, parental attitudes, and perceived burden. This assessment was qualitative and not based on standardized, validated instruments.

Statistical analysis

Statistical analysis was performed using SPSS Statistics version 17.0 (IBM Corp. Released 2008. IBM SPSS Statistics for Windows, Version 17.0. Armonk, NY: IBM Corp.). Quantitative variables were expressed as mean ± standard deviation, while qualitative variables were presented as percentages. Comparisons between groups were performed using the Chi-square test for categorical variables and the independent-samples t-test for normally distributed continuous variables. For each chi-square test, test statistics including degrees of freedom (df) and effect size (Cramér’s V) were reported. A p-value < 0.05 was considered statistically significant.

Ethical considerations

The study respected ethical principles of the Declaration of Helsinki. Informed consent was obtained from parents or legal guardians. All data were anonymized prior to analysis. The study involved human participants using de-identified data. Given the non-interventional nature of the study and in accordance with local regulations, formal ethical approval was not required.

## Results

A total of 171 children with primary enuresis were included in the study, comprising 86 with MPE and 85 with NMPE. Clinical and functional characteristics are summarized in Table 2.

**Table 2:** Clinical and functional characteristics t: independent samples t-test, χ²: chi-square test, * perfect association due to structural differences between groups (0% vs 100%) Subcategories are present for descriptive purposes; statistical comparison applies to the overall variable. MPE: monosymptomatic primary enuresis, NMPE: non-monosymptomatic primary enuresis, SD: standard deviation, FBC: functional bladder capacity

Characteristics	MPE (n = 86)	NMPE (n = 85)	Test statistic	df (degrees of freedom)	P	Effect size (Cramér’s V)
Age (years), mean ± SD	9.12 ± 2.36	7.36 ± 2.02	t = 5.19	169	<0.001	-
Male sex, n (%)	58 (67.4)	38 (44.4)	χ² = 8.97	1	0.003	0.23
Daytime urinary symptoms, n (%)	0	85 (100)	χ² = 171.00	1	<0.001	1.00*
Daytime voiding frequency > 7/day, n (%)	0	73 (85.8)	χ² = 127.47	1	<0.001	0.86
Number of wet nights/ week, n (%)			χ² = 85.67	2	<0.001	0.71
<1 / week, n (%)	4 (4.7)	57 (67.1)	-	-	-	-
1-2 / week, n (%)	33 (38.4)	24 (28.2)	-	-	-	-
≥3 / week, n (%)	49 (56.9)	4 (4.7)	-	-	-	-
FBC, n (%)			χ² = 120.15	1	<0.001	0.84
Normal, n (%)	82 (95.3)	10 (11.7)	-	-	-	-
Reduced, n (%)	4 (4.7)	75 (88.2)	-	-	-	-
Nocturnal polyuria, n (%)	51 (59.3)	1 (1.2)	χ² = 68.25	1	<0.001	0.62
History of urinary tract infection, n (%)	4 (4.7)	28 (32.9)	χ² = 22.49	1	<0.001	0.36
Constipation, n (%)	18 (20.9)	23 (27.0)	χ² = 0.88	1	0.340	0.07
Encopresis, n (%)	4 (4.7)	10 (11.7)	χ² = 2.14	1	0.150	0.11

The mean age was significantly higher in the MPE group than in the NMPE group (9.12 ± 2.36 vs. 7.36 ± 2.02 years; t = 5.19, p < 0.001). A significant male predominance was observed in the MPE group, where boys accounted for 67.4% of the cohort, compared to 44.4% in the NMPE group (χ² = 8.97, p = 0.003; Cramér’s V = 0.23).

By definition, daytime LUTS were absent in all MPE patients and present in all NMPE patients, reflecting the diagnostic criteria for group allocation (χ² = 171.00, p < 0.001; Cramér’s V = 1.00). Similarly, increased daytime voiding frequency was exclusively observed in the NMPE group (85.8% vs. 0%; χ² = 127.47, p < 0.001; Cramér’s V = 0.86).

The number of wet nights per week was also significantly associated with enuresis subtype (χ² = 85.67, p < 0.001; Cramér’s V = 0.71). Patients in the NMPE group more frequently exhibited mild nocturnal symptoms, with 67.1% experiencing fewer than one wet night per week. Conversely, MPE patients more commonly presented with severe forms, with 56.9% reporting three or more wet nights per week compared to only 4.7% in the NMPE group.

FBC was significantly reduced in the vast majority of NMPE patients (88.2%), whereas it remained normal in 95.3% of MPE patients (χ² = 120.15, p < 0.001), demonstrating a strong effect size (Cramér’s V = 0.84). In contrast, nocturnal polyuria was significantly more frequent in the MPE group (59.3% vs. 1.2%; χ² = 68.25, p < 0.001; Cramér’s V = 0.62).

Regarding comorbidities, a history of urinary tract infection was significantly more common among children in the NMPE group than those in the MPE group (32.9% vs. 4.7%; χ² = 22.49, p < 0.001; Cramér’s V = 0.36). No statistically significant differences were found between groups regarding constipation (20.9% in MPE vs. 27.0% in NMPE; χ² = 0.88, p = 0.340; Cramér’s V = 0.07) or encopresis (4.7% in MPE vs. 11.7% in NMPE; χ² = 2.14, p = 0.150; Cramér’s V = 0.11), with small effect sizes indicating weak associations. Psychosocial burden was high in both groups, including parental attitudes and psychosocial factors, with no statistically significant differences between MPE and NMPE (Table 3).

**Table 3:** Psychosocial burden in MPE and NMPE MPE: monosymptomatic primary enuresis, NMPE: non-monosymptomatic primary enuresis

Parental attitudes	MPE (n = 86)	NMPE (n = 85)	χ²	df (degrees of freedom)	P	Effect size (Cramér’s V)
Nocturnal awakening to void, n (%)	53 (61.6)	54 (63.5)	0.06	1	0.79	0.02
Corporal punishment/humiliation, n (%)	34 (39.5)	31 (36.5)	0.17	1	0.68	0.03
Maternal perceived burden, n (%)	69 (80.2)	58 (68.2)	3.22	1	0.07	0.13
Psychological factors						
Social withdrawal, n (%)	46 (53.5)	48 (56.5)	0.15	1	0.69	0.03
Shame, n (%)	38 (44.2)	35 (41.2)	0.15	1	0.69	0.03
Feelings of inferiority, n (%)	25 (29.1)	33 (38.8)	1.81	1	0.18	0.10
Avoidance of sleeping outside home, n (%)	26 (30.2)	27 (31.8)	0.04	1	0.82	0.02

In terms of parental management and attitudes, nocturnal awakening to void was reported in 61.6% of MPE patients and 63.5% of NMPE patients (χ² = 0.06, p = 0.79; Cramér’s V = 0.02). Corporal punishment or humiliation was also comparable between groups (39.5% vs. 36.5%; χ² = 0.17, p = 0.68; Cramér’s V = 0.03). Maternal perceived burden was more frequent in the MPE group (80.2% vs. 68.2%), but this difference did not reach statistical significance (χ² = 3.22, p = 0.07; Cramér’s V = 0.13).

Similarly, psychological factors did not differ significantly between groups. Social withdrawal was observed in 53.5% of MPE patients and 56.5% of NMPE patients (χ² = 0.15, p = 0.69; Cramér’s V = 0.03). Feelings of shame were reported in 44.2% and 41.2% of patients, respectively (χ² = 0.15, p = 0.69; Cramér’s V = 0.03). Feelings of inferiority were slightly more frequent in the NMPE group (38.8% vs. 29.1%), without statistical significance (χ² = 1.81, p = 0.18; Cramér’s V = 0.10). Avoidance of sleeping outside the home was comparable between groups (30.2% vs. 31.8%; χ² = 0.04, p = 0.82; Cramér’s V = 0.02). Overall, no significant associations were found between enuresis subtype and psychosocial variables, with consistently small effect sizes indicating weak relationships.

## Discussion

This study demonstrates that MPE and NMPE represent two distinct clinical phenotypes of primary enuresis with different underlying mechanisms. NMPE was significantly associated with younger age, daytime urinary symptoms, and reduced bladder capacity. These findings are consistent with previous ICCS-based studies, particularly those by Nevéus et al. and Austin et al., which describe NMPE as primarily related to bladder dysfunction and impaired storage capacity [1,2].

Although constipation was more frequent in the NMPE group, this association did not reach statistical significance. This finding contrasts with several previous studies and may be explained by sample size, underreporting, or differences in diagnostic criteria [15,16]. This finding emphasizes the need for systematic and standardized assessment of bowel function in future studies. In contrast, MPE was strongly associated with nocturnal polyuria, supporting the role of altered circadian vasopressin secretion and impaired nocturnal urine concentration [6,7,17].

Our findings are consistent with recent reviews highlighting that MPE is primarily associated with nocturnal urine overproduction, whereas NMPE is associated with bladder dysfunction [18,19]. Children with MPE had more frequent nocturnal enuresis episodes despite normal bladder capacity. This supports the hypothesis that excessive nocturnal urine production exceeds bladder storage capacity during sleep. Conversely, children with NMPE exhibited fewer wet nights but more daytime symptoms, possibly reflecting adaptive voiding behavior and increased daytime bladder activity.

Psychosocial burden was high in both groups, consistent with previous studies demonstrating significant emotional and behavioral impacts [4,5,20]. Behavioral interventions remain a cornerstone of management, particularly in NMPE [21], whereas pharmacological treatments, such as desmopressin, are more effective in MPE [17,22].

Current international guidelines recommend individualized treatment strategies based on enuresis subtype [23,24]. These findings are consistent with current ICCS-based literature and reinforce the importance of distinguishing between enuresis subtypes for optimized management. Future studies incorporating multivariate models and standardized psychosocial assessment tools are needed to better define predictive factors and improve individualized management strategies.

Limitations

This study has several limitations. First, it was conducted in a single tertiary center, which may limit generalizability. Second, the absence of multivariate analysis prevents identification of independent predictors and may have led to residual confounding, thereby limiting the strength of causal inferences. Third, psychosocial assessment was based on non-standardized qualitative interviews, which may introduce subjectivity and reporting bias. Finally, reliance on parental reporting may have led to recall bias.

Strengths

Strengths of this study include the use of ICCS-standardized definitions, a relatively large sample size, and a seven-day voiding diary, which allows objective functional assessment [12,19].

Clinical implications

Differentiation between MPE and NMPE is essential for tailored management, as treatment for MPE primarily focuses on addressing nocturnal polyuria, often with desmopressin. In contrast, NMPE management emphasizes bladder rehabilitation and treatment of associated daytime LUTS.

## Conclusions

MPE and NMPE represent distinct but overlapping clinical entities with different underlying mechanisms. NMPE is primarily associated with bladder dysfunction and daytime symptoms, whereas MPE is mainly related to nocturnal urine overproduction. However, the significant psychosocial burden observed in both groups highlights that enuresis should not be considered a benign condition. A structured clinical evaluation that combines voiding diaries and symptom assessment remains essential for guiding individualized, phenotype-based management strategies. Future studies including multivariate analysis are needed to identify independent predictors.
